# Constipation and Other Disorders of the Large Intestine; Treatment by Olive Oil Enemata1Read before the Journal Club, April 28, 1893.

**Published:** 1893-06

**Authors:** Wilhelm Fleiner, G. A. Himmelsbach

**Affiliations:** Heidelberg; Clinical Instructor in Medicine, University Dispensary, Buffalo, N. Y.


### CONSTIPATION AND OTHER DISORDERS OF THE LARGE INTESTINE; TREATMENT BY OLIVE OIL ENEMATA.[1]

BY PROF. DR. WILHELM FLEINER, Heidelberg.

(Translated from *Berliner Klinische Wochenschrift.*)

BY G. A. HIMMELSBACH, M. D.,

Clinical Instructor in Medicine, University Dispensary, Buffalo, N. Y.

IN ALL cases of constipation demanding therapeutic treatment, we, as practicing physicians, for obvious reasons aim to accomplish one purpose, that is, to perfect an evacuation of the bowels under most all circumstances. Here I wish to state that very often we meet with two forms of constipation, which, however, are entirely different as to their etiology, and present vastly different appearances. Notwithstanding this they are frequently mistaken one for the other; they, therefore, deserve distinct treatment. I refer to *atonic* and *spastic* constipation.

Atonic constipation is the result of insufficient and too slow peristalsis, a disease, when in young people, is indicative of sedentary habits, and in older people it is usually the consequence of a general atrophy, though very often it is the result of a wasted or atrophic condition of the alimentary tract only.

From this form of constipation are developed a variety of manifold bodily ailments, some of them of little concern; some, however, are very aggravating; and still many people suffering thus may reach a good old age, and enjoy comparatively good health. To produce a free evacuation many people need nothing but dietetic remedies, such as very ripe fruit, honey, cornmeal, coarse rye bread, sauerkraut, and sour milk, or some gentle purgative, which may be used now and then, at varying intervals, in small doses, for many years in succession. To these mild laxatives belong, in first order, rhubarb for adults and manna for children; besides these, tamarind, cascara sagrada, and alöes, in small doses. Many use injections of water, or bathing the parts with cold water. I must also mention the use of massage to the abdomen, (sanitary) gymnastics, and electrical applications to the abdomen.

In all such cases we do not deal with a really diseased body, but only with a functional disorder. Nevertheless, atony may develop

1. Read before the Journal Club, April 28, 1893.

into paralysis (or paresis) by extreme distention of some portion of the intestinal tract caused by retarded fecal matter for several days in succession, or even by gases, the result of which often leads to a catarrhal condition of the mucosa. This last disorder can be seen by examination of the stools, which will be found to be intermixed with slimy matter (mucus); this shows the seat of the disease in the colon, sigmoid flexure, or rectum. In all such cases the stools are drier and more solid than usual, and consist in compressed, irregular, hard lumps (scybala), which still show marks or impressions of the haustris in the colon. These scybala are not formed in the rectum or the flexure, but usually before they move from the colon, or even in the cecum. In such cases the rectum may be free from fecal matter, and an injection of water not high enough will have no effect in dislodging the hard, dry lumps, and not until peristalsis is excited, will the scybala lying in or near the colon be brought down.

In atonic constipation, the abdomen is mostly swollen, but even and smooth in all its parts, but when there is only partial atony of the alimentary canal, and the colon becomes filled with gases and fecal matter, then the abdomen is only partly swollen and quite uneven. Partial atony can be more easily recognized when the abdominal wall is thin and flabby than when the wall is thick and muscular.

This atonic constipation is found in adults as well as younger people, and particularly in girls at the age of puberty ; and, moreover, it is found in children more often than it is believed to be. Kussmaul and myself have investigated and observed a girl, four years old, troubled with atony of the colon, and, besides, almost daily, for months, suffered from stercoral spasms. The fecal matter formed in large lumps, filling the region of the flexura hepatica coli, and pressed against the cecum. Sometimes the swelling disappeared, only to return again and again at varying intervals. After continued observation for some time, we observed cylindrical masses pass from the rectum. This case was cured by the faithful use of massage and the use of oil enemata.

A quite different appearance has that form of constipation, caused by a *spastic* condition of the colon. It manifests itself mostly in nervous individuals and those who are victims of nervous exhaustion, in hypochondriacs, and in women who suffer with uterine disorders.

In treating this form of constipation, caused by contraction of certain parts of the colon, it must not be confounded with muscular spasm of the sphincter ani, which is so often the case with fissures about the anus; when the spastic contraction is above the sphincter, soft matter is retained, and greatly distends the colon. The constipation to which I refer here is due to retarded hard lumps of fecal matter, held in certain parts of the canal, and are retained there by localized contraction of the gut, instead of propelling them along. To illustrate this spastic form of constipation, I will suggest that most interesting form of spasm and constipation which accompanies plumbism—lead colic.

A spastic contraction of the sphincter ani, without even the presence of a fissured anus, may present appearances of constipation. A patient of Prof. Kussmaul was relieved of his constipation, which was the result of a spastic contraction of the sphincter, for which an American physician cut the muscle in two. After a few months, however, the former contraction recurred and, indeed, extended farther up the rectum, and the patient's successful treatment and ultimate recovery will be given later. The sphincter showed plainly a cicatrix and thickening where the incision was made.

The discharges, in these cases, are of a peculiar kind and quite characteristic; thin as a tape-worm or a pencil, sometimes long, stringy, then, again, in small particles, so much so that the quantity seems insufficient; sometimes there are small cylindrical masses, not larger than a hazel nut, and even much smaller. Not long ago I saw dejections consisting of one cylindrical string, the thickness of a finger, to which were attached dark, firm balls, at regular distances.

The round-looking excrements, however, are not only characteristic of spastic constipation, for they are also found in the atonic or paralytic form, when the fecal matter is retained at the haustris. *A constant appearance of these round masses is certainly a sign of spastic constipation.* Physicians, as well as the laity, at the sight of these small globuled dejections in spastic constipation, fear the existence of an organic stenosis, of a fissured stricture, or of a recent new formation within the rectum. These fears will be removed by a careful examination of the rectum. The spastic constipation may, or may not, be accompanied by a catarrh of the canal.

In opposition to the atonic constipation which sometimes appears as a complication to that form of colitis which presents a low, asthenic catarrh, I must mention the colitis existing with spastic constipation, and which may be called an irritable form (of the mucosa). Sometimes it is mistaken for colitis membranosa (croupous), a most aggravating affection of the intestine.

In some cases, we observe that the spasm producing constipation is caused by a chemical, thermal, or even a mechanical irritation of the mucous membrane, with subsequent moderate or intense colitis. To these belong, for instance, all those cases that occur after the eating of richly spiced food (pickles), also of too strong laxatives, and especially of the abuse of too drastic purgatives; also, all those cases that follow the rapid cooling off of the body, by sitting on cold stones or moist ground, or in a draught when in a privy.

Any physician who will take the trouble of regularly examining the stools of his patient, will, to his surprise, find the frequent occurrence of this less known spastic constipation, although the atonic is, by far, the more frequent. There are still other forms of constipation in which we find both symptoms, atony and spasm. These may exist side by side (together); then, again, they may exist at different portions of the canal. In some instances, the lower part of the colon is contracted, while the upper portion is atonic and becomes distended by retarded fecal matter and gases.

Massage is of no use in spastic constipation, and is even more harmful than beneficial. The same is true with faradization of abdominal walls, and also the use of drastic purgatives, all of which irritate the intestinal canal. Warm injections of aromatic infusions are here of greater and better use, including chamomile, peppermint, steranis, etc. Sometimes supporting narcotics, such as hyoscyamus and belladonna, improve the condition.

In colica saturnina, opium is of much value and very effectual; however, more efficacious than anything else is the liberal use of oil enemata. In this paper I propose to show the great importance of the use of large oil injections. This method has been practised for years by Prof. Kussmaul, and, indeed, with flattering results. Many physicians have adopted this method in their practice, and, in this way, its use has become quite general. In connection with Prof. Kussmaul, I followed up this method with great success, and, in compliance with his wish, I call it simply the "Oil Cure." I undertook to learn the action of the oil, physically, chemically, and physiologically, and, having arrived at certain conclusions, I strongly believe in its value, and many affections plainly indicate this form of treatment.

*History of Oil Injections.*—The application of oil by rectal injection is by no means a recent treatment. In ancient times olive oil, more especially than other oils, was considered a most valuable remedy. The olive tree was sacred and an object of antiquity. Also in the middle ages olive oil was highly thought of and in general use.

Theodorus Zuingerns says, in his Theatrum Botanicum, page 60 : "Olive oil is quite generally used ; it induces a movement from the bowels."

Praevotius used nothing but olive oil to excite a motion of the bowels of a nobleman, and used a whole pound of the oil for one injection. When the oil is taken per mouth it also has a very beneficial effect, relieving the griping, soothing and healing the internal parts.

Oil enemata are used at the present time in many places. In the clinic at this place pure olive oil is used for injections when the case indicates it. Prof. Erb used it for many years, giving one cupful of the oil, particularly after the use of opium for several days in typhlitis stercoralis.

The beneficial effect of small doses of the oil was known long ago, when one tablespoonful was injected for irritable hemorrhoids, fissures, and rhagaden ani ; also in proctitis and ulcers of the rectum.

Exceptionally do we find the use of oil in large injections, mentioned in modern literature or recent text-books. Professor Kussmaul's attention was first attracted to the use of oil in large injections by a case of the late Dr. Reyher, of St. Petersburg. Through him he learned that large injections of oil softened the solid fecal matter and quieted any irritation of the mucosa. These views are of considerable value and will be referred to later.

*Technique of Oil Injections.*—In order to carry the oil as far up into the colon as possible, from 400 to 500 c. cm. of oil should be used ; the patient is placed upon his back, his hips lying upon a hard cushion about 20 to 25 c. m. high. This should be covered with an oil cloth to prevent the soiling of other things about. This position removes all pressure from the pelvic organs, besides having somewhat of an aspirating power (Hegar).

In order to avoid thermal and mechanical irritation of the intestinal mucosa, which would tend to interrupt peristalsis, the oil used must be raised to the body temperature, and must be introduced slowly and gently, with as little pressure as possible ; pressure not to exceed 50 cm. An ordinary irrigator (fountain syringe), filled with oil, and provided with a guttapercha tube, long enough to answer the purpose, should be used. The instrument for introduction into the bowel should be made of hard rubber, bone, or glass, of the thickness of a finger, very smooth, and having an olive-shaped bulbous tip.

The introduction of such an instrument through the anus is very easy, and safer than a thinner one. The olive tip is never caught in the folds or pockets of the mucous membrane ; it glides smoothly over all without hindrance. The introductory piece should be separate, and not a part of the bulk of the instrument, because evil results are sometimes obtained when the instrument is made in one piece. In order to interrupt the stream and protect the rectum from hash manipulation, an elastic rubber tube should be joined to the instrument. The old style instruments (syringe) are very dangerous, and have caused an everlasting ailment and even death. Much to our distress, we still find many families possessing these old-fashioned bulky instruments, and use them indiscriminately among adults and children. Those instruments with pointed mouth-pieces are dangerous; they emit air into the canal. It is not necessary to have instruments with long mouthpieces to carry the oil up high, as long as the sphincter functionates properly, though there may be instances, when stenosis exists, where longer mouthpieces are of value, in order to pass the stricture.

Costiveness of long duration produces ileus ; the sphincter ani becomes so flabby, relaxed, it offers no hindrance to the introduction of a finger or a tube (Kussmaul). But, even in such cases, it is generally a difficult matter to introduce the tube as far as the lowest part of the sigmoid flexure, and beyond this point *cannot be reached* [(?) (translator)]. The flexure certainly can be pushed up by a hard tube, but, by doing so, there is danger of penetrating the walls of the canal, which is distended to its utmost. Simon has called attention to this fact. The oil flows in slowly, and the tube is advanced cautiously. It will take, at least, fifteen to twenty minutes to introduce from 400 to 500 c. cm. of oil. During this time the oil rises in the colon to a considerable height, quite difficult to define, for only in rare cases is the oil heard to splash in some portions of the canal. In a case of dilatation of the colon ascendens this condition was clearly demonstrated, and, for some days after the oil injections, Prof. Kussmaul and myself could clearly detect the succussion of the oil in the ileo-cecal region.

I hardly think that a single application of a large oil injection is capable of being carried to the cecum, even if we call upon gravitation for assistance, by changing the positions of the patient. We must not feel satisfied that the bowel has received the maximum effect of the oil, even if the dejections have the appearance of fecal matter, commonly found in the small intestine, or even if they contain much bile-coloring matter. We must, therefore, conclude that in order that the oil may produce its effect in the cecum, it must be given every day for several days. Sometimes the end is accomplished the second day; more often, however, it requires three or more days.

Having accomplished a thorough evacuation, the oil injections need not be repeated every day, but only at intervals, also the quantity of oil can be reduced to 250 or 300 c. cm. In local affections of the colon a much smaller quantity of oil will suffice to empty the rectum and sigmoid flexure. A patient is able to take the smaller quantities himself by using an instrument, holding from 100 to 150 c. cm., provided with an olive shaped tip (mouthpiece); with children, from 30 to 50 c. cm. is sufficient.

In these cases an elevation of the hips is not necessary; it requires simply lying upon the back or upon one side. After an application the instrument should be carefully cleansed; water will not do, but with very little difficulty a little alcohol will remove every particle of oil (G. Quincke).

*Some Practical Observations about the Action of the Oil.*—Clinical observations teach us that a discharge of fecal matter does not immediately follow an injection of oil, when given according to the above rules. It takes one, two or more hours before the first act of defecation—some patients even retain the oil all night—and not until just after the regular morning evacuation, will the oil be seen to come too.

Sometimes in cases where the canal is impacted with hard fecal matter, in order to effect an evacuation within three or four hours, it will be necessary to follow the oil injection by one of water. The fecal matter in the bowel, which is dry and hard, becomes coated with oil after the first injection, and when the oil is retained long enough the excrements become more or less softened.

With the act of defecation a part of the oil will also be seen to pass, and a part of the oil will be retained in the canal. Injected oil will not leave the alimentary canal at once, but, on the contrary, it takes several days for it all to pass away. It usually comes away mixed with the feces, but sometimes as pure oil, accompanied by gases. Not infrequently from five to ten days elapse before the oil has been entirely removed, and according to the testimony of one of our attendants, it remained in the bowel fourteen days.

When the oil is passed, after retention in the bowel for some time, it shows peculiar changes; most often is the color of the oil changed from a dark yellow to an olive green. Besides the fecal smell, there is sometimes a sour odor and, chemically speaking, a higher degree of acidity, about which I shall refer to later.

When the bowel is greatly filled with fecal matter, it is necessary to give an injection of oil for several days in succession. In such cases after two or three days the stools will be found to be of a soft, doughy consistency, in consequence of the intermixing of the oil, finally, the passage of a stool very thin and doughy, often bile-stained; this condition of the stool is a positive indication that the maximum effect of the oil has been obtained.

Upon the introduction and advancement of the oil, the patient, as a rule, feels nothing. However, after a shorter or longer time, many experience a peculiar sensation in the alimentary canal, which they define by saying "The oil is searching the canal." This sensation increases with nervous individuals so much so, that when the injection is given at night, even their sleep becomes restless. During this time much gas is passed per bowel. People who are troubled with such sensations do well by lying upon their back, as this position has been found to give the desired relief. Then again with others, a full, free evacuation will only give relief; should the distress continue, an injection of warm infusion of steranis will be found effectual.

Doubtless these subjective symptoms (sensations) are the effects of the oil in the canal and the dissolved, softened particles of excrement with the chemical changes resulting; perhaps also by the advance of the oil in the colon and the irritation caused by it.

*Selection of the Different Kinds of Oils.*—The proper selection of the oil is of importance. These oils are very different according to the quality and chemical composition; it therefore happens sometimes that the physician and patient are greatly disappointed.

In some instances the patient complains of a burning sensation about the anus, immediately after the application; this may also be in the rectum or even higher up in the abdomen. As a consequence, they cannot retain the oil, but discharge it at once with more or less force. This condition can only be checked by injections of lukewarm water, infusions of chamomile, or steranis; in other cases, there is pinching and griping, with frequent discharges of gases and slimy matter; many patients, therefore, refuse to take a second injection until they are assured as to the cause of the first trouble. A careful investigation of this unhappy result leads us to conclude that neither the method nor manner of application was the cause, but especially the quality of the oil, which is often degenerated. This also induced me to search more carefully into the manufacture of these oils. Zuingern's remarks in the *Theatrum Botanicum* are very interesting. "It cannot be denied that in oils there exists a fatty sweetness, also an alkaline salt, which has the power of dissolving all metals, except gold; for that reason such oil should not be used for injuries to the nerves or bones, unless it is boiled, so that the acids and alkalies are destroyed (decomposed)."

Even today the quality of the oil varies according to its method of manufacture. The finest and purest oil is produced by grinding the olives between two millstones. The compressed cake (residue) is then mixed with warm water and again subjected to pressure; the oil now expressed is a poorer quality, but still a salable product for food, olio-lavato. Other fruits of less value produce the huile fermentée, and the least valuable is the olio-inferno (huile d'enfer).[1]

For internal use only the finer grades of olive oil are worthy of consideration. They should contain triolein, 75 per cent., glycerinäther, 25 per cent. The palmitin and the arachïnsaüre congeal the oil. In oils of poorer quality the palmitin and arachin are found in larger proportions. In consequence of the presence of these substances, the oil congeals, or thickens, at about 10° C., while with finer grades of oil, where the tri-palmitin is excluded, the oil will not congeal until 6° C. is reached.

The composition of these oils is greatly affected by climate and soil in which the trees grow, at least the volatile (free) acids are largely dependent upon these. For instance, the California olive oil[2] contains a larger proportion of volatile acids and are, therefore,

1. Flückiger, *Pharmaceut. Chemie.*, Berlin, 1888, II., p. 184.

2. Moerk: Zur Kenntniss des Olivenöls verschiedener Herkunft. Jahresberichte über die Forschritte der Pharmacie, von 1889.

very changeable, much more so than with the Italian, French, Sicilian, or Spanish oils.

With the exception of the finest grades of oil, olive oil is generally mixed with rüböl (brassica rapa), möhnöl (papaver somniferum), cotton seed oil (gossypium herbaceum), sesamöl (sesamum orientale), sunflower seed oil and other cheaper oils. The oily substance found in the dark and light mustard contains sulphur. Besides this, it sometimes contains acid sulphuricum, potassium or sodium ; these are removed by a process of purification. These adulterations of the oil can be determined according to Flückiger, when the total quantity exceeds that of the pure oil by from five to ten per cent., and according to E. Schmidt, from ten to fifteen per cent.

After long exposure to light, olive oil loses its normal color, and becomes whitish. Copper restores the color again and sugar of lead brings back its sweet taste. The addition of one-half per cent. of absolute alcohol preserves the oil and prevents its becoming rancid.

From these statements we are convinced that many oils are modified for the sake of commercial gain. Rotten oils are improved by the addition of certain chemicals, or pure oils are mixed the with other worthless oils. The use of these poor oils will irritate mucous membrane, instead of mitigating and soothing any soreness or irritation. We must, therefore, use the best and purest oils.

The first expression of both the möhnöl (ol. papaveris) or sesamöl are quite equal to a good grade of olive oil. A degree of acidulation of from forty to fifty-five is well borne by the intestines. Oil from the second expression is not as good as the first and should not be given by any physician.

In commerce when we ask for olive oil we are supplied with ol. papaveris or ol. sesamum orientale ; we should, therefore, always ask for the first expressed oil.

Should any irritation of the intestinal tract arise, as the result of an oil injection, the cause will be found to be in the quality of the oil used.

---

Cool Baths in Typhoid.—Dr. Juhel-Rénoy believes that the introduction of the cool bath into the hospital treatment of typhoid fever has reduced the mortality at least five per cent., and cites figures in support of his opinion.—*American Therapist.*

